# Editorial: Molecular diagnostics for cardiovascular rare disease

**DOI:** 10.3389/fmolb.2026.1822197

**Published:** 2026-03-17

**Authors:** Emanuele Bobbio, Martina Caiazza, Giuseppe Limongelli, Francesco Loffredo

**Affiliations:** 1 Inherited and Rare Cardiovascular Diseases, Department of Translational Medical Sciences, University of Campania ‘Luigi Vanvitelli’, Monaldi Hospital, Naples, Italy; 2 Departments of Cardiology, Sahlgrenska University Hospital, Gothenburg, Sweden; 3 Department of Molecular and Clinical Medicine, Institute of Medicine at Sahlgrenska Academy, University of Gothenburg, Gothenburg, Sweden; 4 Institute of Cardiovascular Science, University College London, London, United Kingdom; 5 Department of Translational Medical Sciences, Section of Cardiology, University of Campania Luigi Vanvitelli, Naples, Italy; 6 Vanvitelli Cardiology and Intensive Care Unit, Monaldi Hospital, Naples, Italy

**Keywords:** cardiovascular genetics, Genotype–phenotype, molecular diagnostics, precision cardiology, rare diseases, structural variants

Rare cardiovascular diseases are often diagnosed late, not because they are clinically silent, but because their biological signals are fragmented across different diagnostic layers. Genomic variation may be present without clear functional consequences, imaging abnormalities may overlap with common age-related changes, and clinical phenotypes may only fully emerge years after the initial molecular insult (Ackerman et al., 2011). In this context, the central challenge is no longer the lack of diagnostic tools, but the difficulty of integrating them into a coherent diagnostic strategy (Hershberger et al., 2018).

The contributions gathered in this Research Topic address this challenge from complementary angles, collectively arguing that precision in rare cardiovascular disease depends on interrogating the right biological layer at the right moment. Rather than proposing a single “best” technology, these studies show how genome structure, transcript-level function, quantitative imaging phenotypes, and genotype-informed clinical evaluation can converge to reduce diagnostic uncertainty and improve interpretability.

One persistent source of uncertainty in congenital heart disease lies in genomic architecture. Despite widespread use of chromosomal microarray analysis and sequencing-based approaches, a substantial proportion of patients, particularly those with complex phenotypes such as heterotaxy, remain without a molecular diagnosis (Glessner et al., 2014). Structural variants, including balanced rearrangements and complex genomic events, represent a plausible contributor to this missing heritability but are incompletely captured by standard methods. Optical genome mapping has emerged as a next-generation cytogenetic approach capable of genome-wide structural variant detection with high resolution and without reliance on amplification (Neveling et al., 2021). Its clinical relevance has been demonstrated in several disease settings, supporting its role as a complementary diagnostic layer rather than a replacement for sequencing.

Within the present Research Topic, Min et al. apply optical genome mapping to patients with congenital heart disease and heterotaxy whose prior genetic testing had been inconclusive. By integrating structural variant data with existing genomic information, they identify clinically relevant rearrangements affecting known disease-associated genes, as well as candidate loci that merit further investigation. The broader implication of this work is conceptual: unresolved cases may not reflect “negative genetics,” but rather incomplete interrogation of genome structure.

Even when sequence variation is identified, diagnostic ambiguity often persists. Variants affecting splicing, transcript stability, or allele-specific expression may remain classified as uncertain when evaluated at the DNA level alone. Transcriptome-informed diagnostics have therefore gained increasing attention, with RNA sequencing shown to improve diagnostic yield and clarify pathogenic mechanisms across Mendelian diseases (Cummings et al., 2017). This functional dimension is particularly relevant for large, multi-exon genes with complex expression patterns.

Irshaid et al. address this challenge in *FBN1*, the gene encoding fibrillin-1 and the principal molecular determinant of Marfan syndrome, by developing a targeted whole-blood RNA sequencing assay covering the full coding region of *FBN1*. Applied to families with suspected Marfan syndrome, the assay not only detects pathogenic variants with high sensitivity but also reveals their transcriptional consequences, including aberrant splicing and RNA polymerase slippage. This functional evidence directly supports diagnostic confidence in a condition where accurate classification has profound clinical implications for aortic surveillance and prophylactic intervention, as emphasized by the revised Ghent nosology (Loeys et al., 2010). More broadly, this work illustrates how RNA-based approaches can act as a bridge between genotype and mechanism, transforming uncertain variant calls into clinically meaningful diagnoses.

Molecular diagnosis, however, does not end at variant interpretation. It must ultimately inform risk stratification and monitoring at the organ level. Fabry disease exemplifies this translational challenge. Although its molecular basis is well defined, early cerebrovascular involvement can be difficult to distinguish from common vascular changes using conventional imaging. Prior studies have described characteristic posterior circulation involvement, including basilar artery remodeling, but these findings have rarely been integrated into a unified diagnostic framework (Manara et al., 2017).

Roh et al. propose such a framework by combining microvascular and macrovascular brain MRI markers in genetically confirmed Fabry disease. Using high-resolution vessel wall imaging, they demonstrate that perivascular space burden and basilar artery remodeling are coupled in Fabry disease but not in matched controls, suggesting a disease-specific pattern of vascular degeneration. This approach reframes advanced imaging as a quantitative downstream phenotype of molecular pathology positioned between genotype and clinical events, and potentially valuable for early diagnosis and longitudinal monitoring.

Finally, molecular diagnostics should not be viewed solely as a means of etiologic classification. Their clinical value also lies in identifying modifiable contributors to disease expression. Pediatric primary cardiomyopathies are rare, genetically heterogeneous, and associated with high morbidity and mortality, prompting major societies to emphasize systematic phenotyping and genetic evaluation as the foundation of care (Lipshultz et al., 2019). Yet even in genetically defined disease, biological modifiers may influence severity and reversibility.

In this context, Jiang et al. explore the relationship between genetic findings and electrolyte and amino acid pathways in children with primary cardiomyopathies. In a genetically characterized cohort, they report improvements in cardiac function among children with dilated cardiomyopathy treated with calcium supplementation. While exploratory, these observations resonate with earlier reports of hypocalcemia-induced cardiomyopathy reversible with calcium replacement (Sanyal and Raychaudhuri, 2013). The implication is not that metabolic correction substitutes genetic diagnosis, but that molecularly characterized patients may still benefit from systematic evaluation of biologically plausible, potentially reversible modifiers, particularly in early life.

Taken together, the studies in this Research Topic articulate a coherent vision of molecular diagnostics in cardiovascular rare disease. Diagnostic precision is achieved not by accumulating tests, but by aligning technologies with biological questions: genome structure when sequence is insufficient, transcript function when interpretation is uncertain, quantitative imaging when phenotype lacks specificity, and genotype-informed evaluation when therapeutic opportunities exist. In rare cardiovascular disease, molecular diagnostics is no longer about naming the disease, but about revealing which biological layer must be interrogated to understand it.
